# Development of a lytic peptide derived from BH3-only proteins

**DOI:** 10.1038/cddiscovery.2016.8

**Published:** 2016-03-07

**Authors:** Q Liu, H Zhao, Y Jiang, M Wu, Y Tian, D Wang, Y Lao, N Xu, Z Li

**Affiliations:** 1 School of Chemical Biology and Biotechnology, Peking University Shenzhen Graduate School, Shenzhen, China; 2 Shenzhen Key Lab of Tissue Engineering, The Second People's Hospital of Shenzhen, Shenzhen, China; 3 School of Pharmacy, Shanghai University of Traditional Chinese Medicine, Shanghai, China; 4 Key Lab in Healthy Science and Technology, Division of Life Science, Tsinghua University Shenzhen Graduate School, Shenzhen, China

## Abstract

Despite great advances in cancer therapy, drug resistance is a difficult hurdle to overcome that requires development of anticancer agents with novel and effective modes of action. In a number of studies, lytic peptides have shown remarkable ability to eliminate cancer cells through a different way from traditional treatments. Lytic peptides are positively charged, amphiphilic, and are efficient at binding and disrupting the negatively charged cell membrane of cancer cells. In this study, we described the anticancer properties of a lytic peptide that was developed on the basis of the alignment of amphiphilic BH3 peptides. Our results demonstrated that the positive charge and conformation constraint were favourable for efficient cancer cell elimination. Artificial BCL-2 homology 3 peptides (ABH3) exhibited effective anticancer effects against a series of cancer cell lines *in vitro* and in HeLa human cervical tumour xenografts *in vivo*. ABH3 induced cell death in an apoptosis-independent manner through the lytic properties of the peptide that caused disruption of cell membrane. Our results showed that charge tuning and conformation constraining in a lytic peptide could be applied to optimise the anticancer activity of lytic peptides. These results also suggest that ABH3 may be a promising beginning for the development of additional lytic peptides as anticancer reagents.

## Introduction

Despite significant strides in cancer treatment strategies, drug resistance in cancer is one of the leading causes of death worldwide.^1,2^ There is an urgent need for the development of novel anticancer strategies. Cytolytic peptides emerge as a new class of promising anticancer reagents owing to their lytic nature.^3–5^

Most lytic peptides are cationic and amphiphilic in nature. Their mechanism of action is primarily characterized by electrostatic interactions with the plasma membrane, followed by membrane disruption and pore formation, which can lead to rapid necrotic cell death.^6–8^ However, applications of lytic peptides are largely limited by non-specific toxicity, such as haemolysis, towards normal cells.^9^ Furthermore, some solid tumours are insensitive to known lytic peptides.^4^ In order to utilise the properties of lytic peptides for therapeutic use, the development of a tool box of engineered lytic peptides with anticancer activities is essential. Wimley *et al.*^10,11^ tuned the conformation of lytic peptides to modulate their activities, thereby identifying gain-of-function or loss-of-function variations.

In this study, we report an artificial 18-mer BCL-2 homology 3 peptide (ABH3) that was engineered from the original BH3 sequence *via* charge tuning and conformation constraining. ABH3 exhibited wide-spectrum growth inhibition over various cancer cell lines *in vitro* and in HeLa human cervical tumour xenografts *in vivo*. These results strongly promote the use of ABH3 as a lytic peptide and serve as a model for the development and optimisation of lytic peptides based on other architectures for use as anticancer agents.

## Results

### BH3 peptides are potential lytic peptides

B-cell lymphoma-2 (BCL-2) family proteins have a pivotal role in regulating cell death.^12–16^ Among them, BCL-2 homology 3 (BH3) only proteins promote apoptosis through anti-sequestration or direct activation of apoptosis effectors.^17–19^ BH3-only proteins are typical amphiphilic peptides with conserved rhythmic hydrophobic and positively charged residues that are consistent with features of other lytic peptides as shown by the alignment of BH3 peptides^20,21^ (Supplementary Figure S1). The BH3-only domain contains roughly 20 amino acids, which matches the thickness of most bio-membranes. We envisioned that the BH3-only proteins might provide an ideal skeleton for the development of novel lytic peptides.

### Conformation constraint and positive charge are favourable for cell-killing properties of ABH3

**Figure 1:**
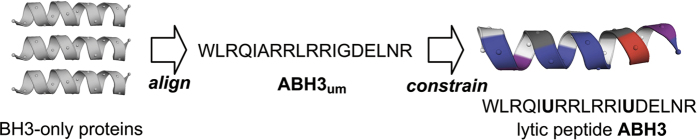
Design of a lytic peptide ABH3 based on BH3-only proteins. (Blue: basic amino acids, red: acidic amino acids; purple: polar amino acids, white: non-polar amino acids; black: Gly or Ala).

To develop our novel peptide, we first engineered an initial peptide derived from the BH3 sequence. During peptide synthesis, we added a Trp amino acid at the N-terminus for facile concentration determination and achieved the unmodified artificial BCL-2 homology 3 peptide (ABH3_um_, Ac-WLRQIARRLR- RIGDELNR-NH2). The helically constrained peptide showed an amphiphilic conformation as shown by the coloured cartoon (Figure 1).

**Figure 2:**
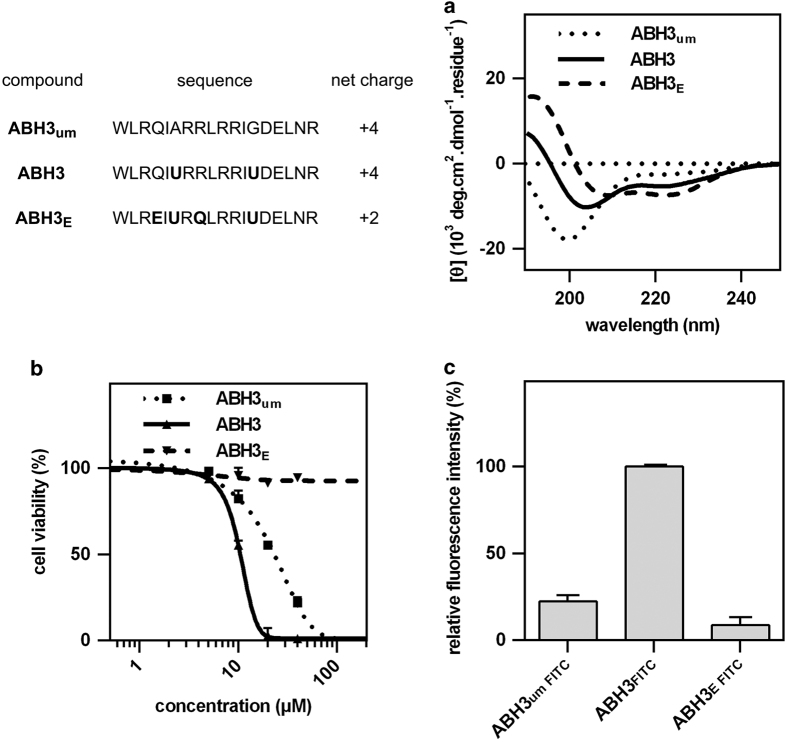
Amphiphilicity and positive charge are required for cell-killing properties of ABH3 peptides. (**a**) CD spectra of ABH3_um_, ABH3 and ABH3_E_. CD spectral measurements were performed in PBS buffer, 10 mM, pH 7.4, 298 K; (**b**) Cell viability of HCT116 cell lines after treatment with ABH3_um_, ABH3 and ABH3_E_ (24 h); (**c**) Cellular uptake of ABH3_um FITC_, ABH3_FITC_ and ABH3_E FITC_, (1 h). Values are expressed as mean (±S.D.) (*n*=3).

ABH3_um_ showed limited helicity in PBS (Figure 2a) according to the CD spectrum and moderate cell-killing activity (IC_50_~30 *μ*M) against HCT116 according to the MTT results (Figure 2b). Conformation constraining has achieved great success in making peptides more effective. These reports prompted us to incorporate conformation constraining amino acids to achieve more helical peptides and test their effects. The Ala and Gly in ABH3_um_ were replaced with a dihedral angle-constraining amino acid, aminoisobutyric acid, to enhance the peptide’s conformation constraint.^22^ The resulting peptide, ABH3 (Ac-WLRQIURRLRRIUDELNR-NH_2_, U=aminoisobutyric acid), showed enhanced helicity and cellular activity (Figures 2a and b). These results indicated that conformation constraining is favourable for enhancing the cytotoxicity of ABH3. However, a less positively charged control peptide, ABH3_E,_ with two amino acid residues altered to Glu (Ac-WLREIURQLRRIUDELNR-NH_2_, U=aminoisobutyric acid) showed higher helicity than ABH3 but negligible effect on cell growth of HCT116 cells (Figures 2a and b). As a result, the combination of conformation constraint and a higher positive charge were favourable for cell-killing activities of ABH3. Besides, cell viability correlated well with the cellular uptake of these peptides as quantified by FACS (Figure 2c). ABH3 exhibited a roughly fivefold increase in permeability compared with ABH3_um_ or ABH3_E_ (Figure 2c). These results illustrated the impact of both of the peptide’s helicity and net charge to their effective penetration and cell-killing. Although ABH3_um_ showed similar cellular penetration with ABH3_E_, their cellular activities are quite different. In summary, this experiment confirmed that the net charge of the engineered peptide has a central role in the cellular activity of lytic peptides and demonstrated that positive charge tuning and conformation constraining were efficient methods for enhancing the cell-killing properties of lytic peptides. These observations could be further applied in lytic peptide development using other peptide skeletons.

### ABH3 induced cell death in an apoptosis-independent pathway

**Figure 3:**
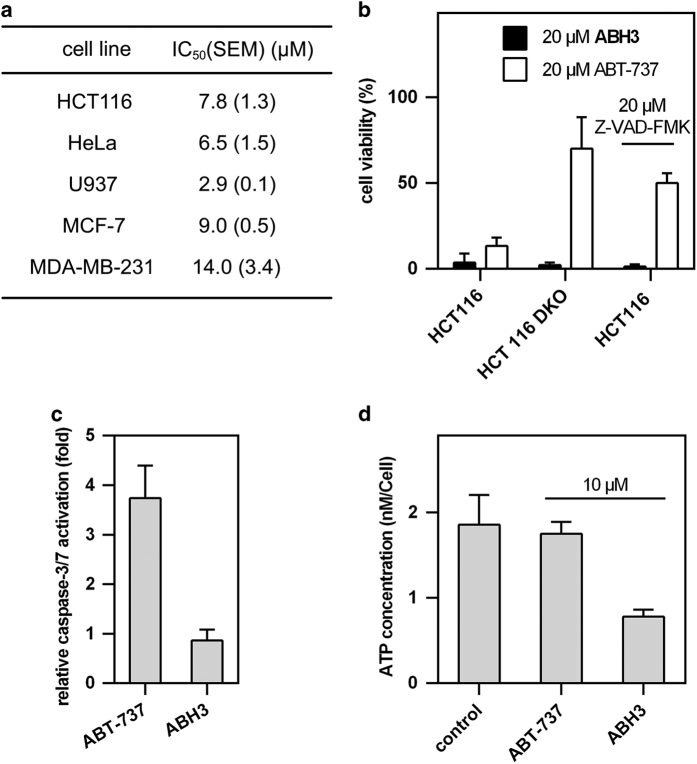
ABH3 induced cell death in an apoptosis-independent way. (**a**) Action of ABH3 on different cancer cell lines (24 h); (**b**) Action of ABH3 lytic peptide and ABT-737 with or without Z-VAD-FMK on HCT116 and the Bak/Bax double knockout HCT116 cell line; (**c**) Caspase activation in HCT116 after treatment with ABH3 and ABT-737 (10 *μ*M, 4 h); (**d**) ATP depletion in HCT116 after treatment with ABH3 and ABT-737 (10 *μ*M, 4 h). Values are expressed as mean (±S.D.) (*n*=3).

In addition to the HCT116 cell line, ABH3 induced efficient cell death in cervical cancer cell HeLa, leukaemia cell U937, breast cancer cell MCF-7 and MDA-MB-231. The IC_50_ of most cell lines (HCT116, HeLa, U937, MCF-7) was less than 10 *μ*M while that of MDA-MB-231 was higher. (Figure 3a).

We conducted several apoptosis-related assays to determine the peptides’ mechanism of action. There were no significant differences between the action of ABH3 on wide-type HCT116 cells and Bak/Bax double knockout HCT116 cells. In contrast, the selective BCl-2 inhibitor ABT-737 showed significant killing efficiency differences in these two cell lines (Figure 3b). In addition, the caspase inhibitor Z-VAD-FMK could not block the cell-killing effects of ABH3 but was able to efficiently diminish the cell-killing effect of ABT-737 (Figure 3b). Furthermore, no caspase-3/7 activation (Figure 3c) and gross ATP depletion was observed in ABH3-treated HCT116 cells (Figure 3d). These observations strongly suggest that ABH3 kills cells *via* an apoptosis-independent pathway in contrast to ABT-737. Notably, ABH3 showed significant advantage over the ABT-737 compound in treating Bak/Bax-deficient cells, which suggests that lytic peptides may be able to overcome drug resistance in cancer therapy.

### ABH3 caused severe membrane disruption

**Figure 4:**
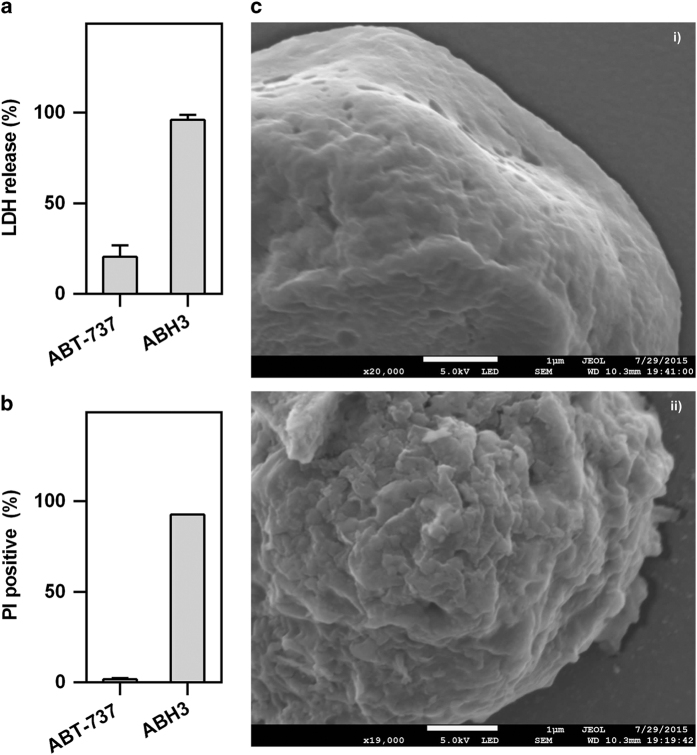
ABH3 induced cell membrane destruction. (**a**) LDH release of HCT116 following treatment of ABH3 or ABT-737 (20 *μ*M, 4 h); (**b**) PI staining of U937 cells following treatment with ABH3 and ABT-737 (10 *μ*M, 0.5 h); (**c**) Scanning electron microscope image of HCT116 upon treatment with ABH3 (10 *μ*M, 1 h), (i) control, (ii) treated by ABH3. Values are expressed as mean (±S.D.) (*n*=3).

Next, we used a lactate dehydrogenase (LDH) release assay to investigate the ability of ABH3 to disrupt cell membranes (Figure 4a). Treatment with ABH3 led to an almost complete loss of membrane integrity. In addition, quick PI staining following treatment of ABH3 also indicated a rapid cell membrane breakage (Figure 4b). Scanning electron microscope observations of the HCT116 cell membranes showed damaged membrane morphology after treatment with ABH3 for 1 h (Figure 4c). These results all demonstrated the ability of ABH3 to drastically disrupt the cell membrane and change the cell permeability.

### ABH3 showed anticancer activity in a xenograft model

**Figure 5:**
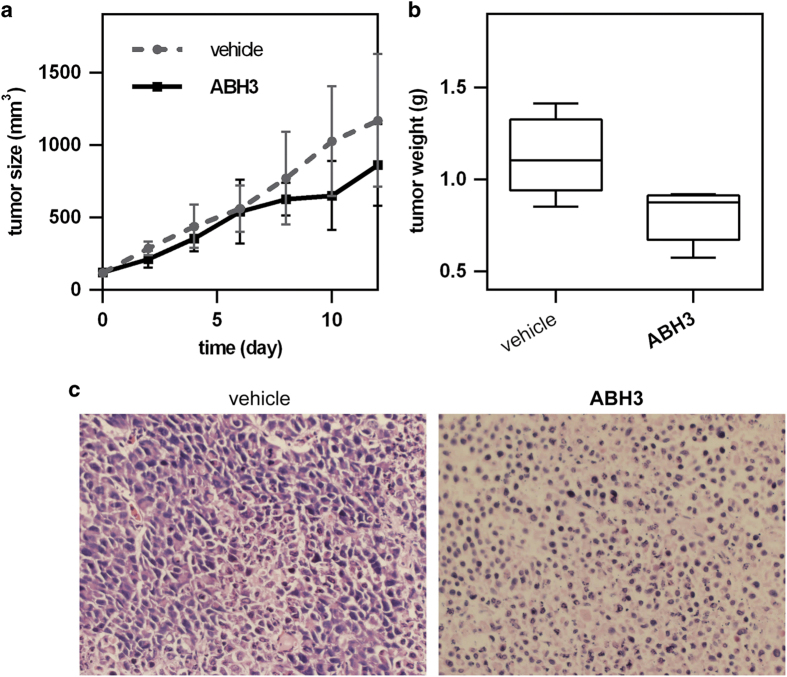
Anticancer efficacy of ABH3 *in vivo.* (**a**) Tumour size of HeLa xenografts. Tumour size was measured by calliper measurements over a period of 2 weeks. (**b**) Tumour weights of HeLa xenografts. Mice were killed and tumours were resected after the final injection. Error bars represent maximum and minimum; boxes represent the upper and lower quartiles and median; (**c**) H&E staining of tumour cross-sections from mice treated with control and ABH3. Scale bar, 20 *μ*m. mean±S.D. (*n*=5).

To study the anticancer activity of ABH3 *in vivo*, we injected nude mice with human cervical cancer cells (Hela) and then administered ABH3 or vehicle control. Tumour volume and weights was significantly reduced after treatment of ABH3 for 12 days compared with vehicle control where tumour volume increased dramatically (Figures 5a and b). Histological stains (H&E staining) showed that the cells were densely packed in the tumour tissue of the control mice but that the cell density was significantly reduced in the tumour tissue of ABH3-treated (Figure 5c). Overall, these results demonstrated that ABH3 has a significant antitumor effect *in vivo*.

## Discussion

Here, we show that an artificial lytic peptide BCL-2 homology 3 peptides (ABH3) derived from BH3-only proteins induces cell death in cancer cells *in vitro* and prevents tumour growth as single agent in an established HeLa human cervical tumour xenograft model. Positive charge tuning and conformation constraining are efficient methods for enhancing the cell-killing properties of lytic peptides as shown by the comparison of ABH3_um_, ABH3 and ABH3_E_. ABH3 induced efficient cell death in colon cancer cells (HCT116), cervical cancer cells (HeLa), leukaemia cells (U937) and breast cancer cells (MCF-7, MDA-MB-231). Unsurprisingly, the several apoptosis-related assays we conducted strongly suggest that ABH3 kills cells *via* an apoptosis-independent pathway. Further, the LDH release, PI positively staining and membrane shrinking shown by scanning electron microscope demonstrate the ability of ABH3 to drastically change the cell permeability of the cells and to rapidly disrupt the cell membrane. Together, lytic peptide ABH3 induces cell death in an apoptosis-independent manner through its lytic nature that causes disruption of cell membrane.

The BH3-only proteins are typical amphipathic helices with conserved hydrophobic and hydrophilic residues. Their BH3 domains contain roughly 20 amino acids, which matches the thickness of most bio-membranes. On the basis of these, we reasoned that BH3-only proteins might provide an ideal skeleton for the development of novel lytic peptides. This preconception is then further verified with experimental results. Modification of BH3 peptides were reported intensively for various biological applications;^21,23–25^ we are the first to develop the ABH3 on the basis of the BH3 peptide as a lytic peptide to induce cancer cell death *via* an apoptosis-independent manner, and ABH3 may be helpful to overcome drug resistance in cancer therapy as shown by the cell-killing effects of ABH3 over HCT116 DKO. This is a new application we developed from an old framework.

Besides applying the amphiphilic nature of BH3 peptides, we introduced charge tuning and conformation constraining in lytic peptide development. The results indicate that these two methods are effective for lytic peptide development as shown by the comparison of ABH3_um_, ABH3 and ABH3_E_. The positive charge was favourable for the membrane adhesion and insertion. The conformation constraint may contribute to the amphiphilicity of peptides as the helical cartoon shown in Figure 1. In addition, the current study has demonstrated that the introduction of a hydrophobic unnatural amino acid has positive effect on cell-killing through conformation constraining.

ABH3 triggered cell death in a wide panel of cancer cell lines including colon cancer, cervical cancer, breast cancer and leukaemia cancer cells. ABH3 showed higher toxicity towards the suspension cancer cell line U937. The sensitivity to U937 was also observed in previous studies on BH3-only peptides and other lytic peptides.^4^ We hypothesised that the suspension nature or membrane composition might explain the discrepancy. The detailed reasons behind the various sensitivity are currently unclear. Intra-tumour administered ABH3 prevented tumour growth as a single agent in an established HeLa human cervical tumour xenograft model. However, non-specific toxicity to normal cell lines (indicated by mouse embryonic fibroblast cells MEF, Supplementary Figure S2, and by haemolysis, Supplementary Figure S3) limited its further *in vivo* application. Besides, peptides, especially cationic ones, may cause immune response when administered systematically. Accordingly, we adopted the intra-tumour administration to avoid non-specific toxicity or unexpected immune response. Furthermore, it is reported that intra-tumour injection of peptides may enhance tumour cell antigenicity in cancer immunotherapy.^26^ To sum up, targeting strategies should be developed to make ABH3 selective.

In summary, we used rational design based on BH3 peptide alignment, conformation constraint and charge tuning to engineer a novel lytic peptide ABH3. We found that conformation constraint and increased net positive charge are both favourable for achieving effective and wide-spectrum cell-killing lytic peptides. ABH3 shows good cancer cell growth inhibition activity both *in vitro* and *in vivo*. The results suggest that ABH3 could be regarded as an anticancer candidate for further development. Non-specific toxicity towards normal cell lines remains the primary shortcoming of ABH3 peptide; further sequence optimisation, incorporation of novel unnatural amino acids, introduction of chemical constraints and conjugation with other peptide sequences for better cancer cell selectivity are currently under investigation in our laboratory.

## Materials and Methods

### Peptide synthesis

MBHA resin, amino acids and other reagents were purchased from GL Biochem (Shanghai, China), Hanhong Chemical (Shanghai, China), Energy Chemical (Shanghai, China) and used as received. N-Methyl-2-pyrrolidone was purchased from Tenglong Logistics (Shenzhen, China). Other solvents were purchased from Cantotech Chemical (Shenzhen, China).

Peptides were synthesised on Rink-amide-MBHA resin using manual Fmoc/t-Butyl solid phase peptide synthesis. Coupling reactions were performed using O-(6-chloro-1-hydrocibenzotriazol-1-yl)-1,1,3,3-tetramethyl-uronium hexafluorophosphate or N-hydroxybenzotriazole/diisopropyl carbodiimde for 3 h with N2 bubbling. FITC labelling was performed on the resin with the solution of fluorescein isothiocyanate (isomer I, 7 eq) and N, N-diisopropylethylamine (14 eq) in dimethylformamide overnight. Final resins were treated with 95% (v/v) trifluoroacetic acid/triisopropylsilane/H2O (95 : 2.5 : 2.5) for 2 h. After air removal of most of the trifluoroacetic acid, products were triturated with hexane/diehyl ether (1 : 2), dissolved in CH3CN/H2O (1 : 9). Crude peptides were purified on RP-HPLC (Shimadzu (Kyoto, Japan), Agilent (Santa Clara, CA, USA) Zorbax SB-Aq: 4.6×250 mm, 220 and 254 nm) and confirmed by Shimadzu LCMS 2020 mass-spectrometer equipped with Agilent Zorbax SB-Aq column.

### Cell viability assay

For adherent cells, cells were plated 5000 per well on 96-well plates in medium containing 10% serum and incubated with serial dilution of peptides or ABT-737 with/without 20 *μ*M Z-VAD-FMK for 24 h. Twenty microliters of MTT labelling reagent were then added to each well and the cells incubated at 37 °C for 4 h. The precipitate was solubilized for 5 min and then quantitated by a microplate reader (Perkin Elmer (Waltham, MA, USA), Envision, 2104 multilabel reader).

For U937 (suspension cell), cells were plated 8000 per well on 96-well plates in medium containing 10% serum and incubated with serial dilution of ABH3 for 24 h. Ten microliters of CCK-8 reagent (Dojindo, Kyushu Island, Japan) were then added to each well and the cells incubated at 37 °C for 1–2 h and then quantitated by a microplate reader.

### Cell penetration

Adherent HCT116 cells were seeded overnight in 12-well plates, and then treated with FITC-labelled peptides (2.5 *μ*M) in serum-free medium for 1 h at 37 °C. The cells were digested by 0.25% trypsin and collected. The cells were washed by PBS for three times further analysed by flow cytometry (BD Science, Franklin Lakes, NJ, USA).

### Caspase-3/7 activation analysis

Cells were plated 10 000 per well on 96-well plates in medium containing 10% serum and incubated with serial dilution of ABH3 and ABT-737 for 4 h. Then caspase-3/7 activation was measured by addition of the caspase-Glo 3/7 chemiluminescence reagent in accordance with the manufacturer’s protocol (Promega, Madison, WI, USA). Luminescence was detected by a microplate reader.

### ATP depletion

Cells were plated 20 000 per well on 12-well plates in medium containing 10% serum and incubated with serial dilution of ABH3 and ABT-737 for 4 h. Then, the media was removed followed by washing with PBS twice. The cells were treated with 100 *μ*l lysis buffer (1 nM phenylmethlysulfonyl fluoride, 0.2 mM sodium orthovanadate and 10 mg/ml aprotinin). Then, the ATP levels were detected in accordance with the manufacturer’s protocol (Promega).

### LDH release

Cells were plated 5000 per well on 96-well plates in medium containing 10% serum and incubated with serial dilution of ABH3 and ABT-737 for 4 h. Then, LDH release was measured by the LDH release kit (Dojindo).

### PI staining

U937 cells were treated with ABH3 (10 *μ*M) in serum-free medium for 1 h at 37 °C. Then, cells were collected by centrifuge and stained with PI solution. The cells were washed by PBS for three times further analysed by flow cytometry (BD Science).

### Electron microscopy

HCT116 cells treated with 10 *μ*M ABH3 were harvested and washed with PBS three times. Cells were fixed with 4% glutaraldehyde in 0.2 M Na-cacodylate buffer, pH 7.4, for 3 h at 4 °C followed by dehydration with graded series of ethanol. After lyophilization, coating was carried out with gold and observed under scanning electron microscope.

### Xenograft tumour model

Four-week-old nude mice were purchased from the Experimental Animal Center of Chinese Academy of Science (Shanghai, China). Approximately 1×10^6^ HeLa cells were injected into the right flank of mice. One week later, mice bearing tumours around 50 mm^3^ in volume were randomly divided into three groups. Mice were administered via intra-tumour injection vehicle control solvent (0.5% DMSO, 0.5% Tween-80 in saline) and ABH3 at the dose of 25 or 50 *μ*g in 200 *μ*l vehicle every day. Tumour size was monitored and measured by caliper measurements over a period of 2 weeks. The volume was calculated using the formula: V=1/2ab^2^ (where a is the largest diameter and b is the smallest diameter). Tumour xenografts were excised, routine fixed, paraffin-embedded and sliced for H&E staining and TUNEL assay.

### Haemolysis assays

Fresh mouse blood was collected in anticoagulant tubes. The red blood cells were washed and diluted to 1×10^7^/ml. Various concentrations of ABH3 were incubated with 50 *μ*l of red blood cells at 37 °C for 1 h. Red blood cells treated with 1% Triton was used as a positive control. The absorbance (540 nm) of the supernatants were measured using a microplate reader.

## Abbreviations

BCL-2: B-cell lymphoma-2
ABH3: artificial 18-mer BCL-2 homology 3 peptide
BH3: BCL-2 homology 3
ABH3_um_: unmodified artificial BCL-2 homology 3 peptide
PBS: phosphate-buffered saline
CD spectrum: circular dichroism spectrum
MTT: 3-(4,5-dimethyl-2-thiazolyl)-2,5-diphenyl-2-H-tetrazolium bromide
ABH3_E_: Glu replaced ABH3
FACS: fluorescence activating cell sorter
LDH: lactate dehydrogenase
PI: propidium iodide
H&E staining: hematoxylin-eosin staining.
