# Supplementary material for: Development of a lytic peptide derived from BH3-only proteins
Source: Cell Death Discov. 2016 Mar 7;2:16008–. doi: 10.1038/cddiscovery.2016.8 (PMC4979451; doi:10.1038/cddiscovery.2016.8)
Supplement: Supplementary Information [file cddiscovery20168-s1.doc]

**Development of a lytic peptide derived from BH3-only proteins**

Qisong Liu^1, 4^, Hui Zhao^1^, Yanhong Jiang^1^, Man Wu^2^, Yuan Tian^1^, Daping Wang^4^, Yuanzhi Lao*^2^, Naihan Xu*^3^, Zigang Li*^1^

1 School of Chemical Biology and Biotechnology, Peking University Shenzhen Graduate School, Shenzhen, 518055, China

2 School of Pharmacy Shanghai University of Traditional Chinese Medicine Shanghai, 201203 (China)

3 Key Lab in Healthy Science and Technology, Division of Life Science, Tsinghua University Shenzhen Graduate School, Shenzhen, 518055, China

4 Shenzhen Key Lab of Tissue Engineering, Shenzhen Second People's Hospital Shenzhen, 518035, China

**Alignment of BH3-only proteins**

**Figure S1** Alignment of BH3-only proteins and the sequence of ABH3_0_, ABH3


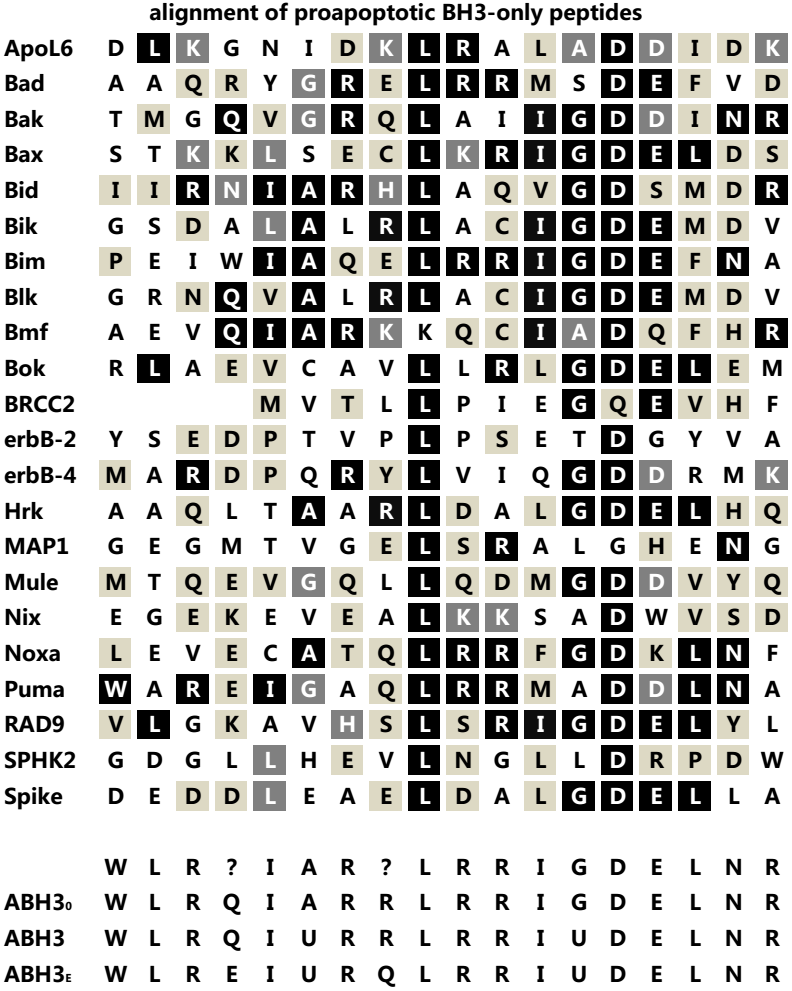


**MTT experiments of MEF**

**Figure S2** MTT results of **ABH3** on MEF cell line

**Figure S3** Hemolysis results of ABH3.

**Peptide synthesis**

MS data collection.

| sequence | calculated mass | found mass |
| --- | --- | --- |
| **BIM SAHB_A_**  Ac-Ile-Trp-Ile-Ala-Gln-Glu-Leu-Arg-cyclo(S5-Ile-Gly-Asp-S5)-Phe-Gln-Ala-Tyr-Tyr-Ala-Arg-Arg-NH_2_ | 2645.4231 | 1324.7[M+2H]  883.3[M+3H] |
| **1** **ABH3_0_**  Ac-Trp-Leu-Arg-Gln-Ile-Ala-Arg-Arg-Leu-Arg-Arg-Ile-Gly-Asp-Glu-Leu-Asn-Arg-NH_2_ | 2361.3730 | 826.5[M+3K] |
| **1_FITC_ ABH3_0_**  FITC-βAla-Trp-Leu-Arg-Gln-Ile-Ala-Arg-Arg-Leu-Arg-Arg-Ile-Gly-Asp-Glu-Leu-Asn-Arg-NH_2_ | 2781.4510 | 928.3[M+3H]  965.9[M+3K] |
| **2 ABH3**  Ac-Trp-Leu-Arg-Gln-Ile-Aib-Arg-Arg-Leu-Arg-Arg-Ile-Aib-Asp-Glu-Leu-Asn-Arg-NH_2_ | 2403.4200 | 840.7[M+3K] |
| **2_FITC_ ABH3_FITC_**  FITC-βAla-Trp-Leu-Arg-Gln-Ile-Aib-Arg-Arg-Leu-Arg-Arg-Ile-Aib-Asp-Glu-Leu-Asn-Arg-NH_2_ | 2823.4980 | 941.9[M+3H]  980.0[M+3K] |
| **3 ABH3_E_**  Ac-Trp-Leu-Arg-Glu-Ile-Aib-Arg-Glu-Leu-Arg-Arg-Ile-Aib-Asp-Glu-Leu-Asn-Arg-NH_2_ | 2377.3455 | 793.9[M+3H], |
| **3_FITC_ ABH3_E FITC_**  FITC-βAla-Trp-Leu-Arg-Glu-Ile-Aib-Arg-Glu-Leu-Arg-Arg-Ile-Aib-Asp-Glu-Leu-Asn-Arg-NH_2_ | 2797.4235 | 1399.6[M+2H],  933.3[M+3H], |
